# Supplementary material for: Predicting visual function by interpreting a neuronal wiring diagram
Source: Nature. 2024 Oct 2;634(8032):113–23. doi: 10.1038/s41586-024-07953-5 (PMC11446822; doi:10.1038/s41586-024-07953-5)
Supplement: Supplementary file 6 — CRF and ERF predictions for individual TmY4 and TmY9 cells. Analogous to Supplementary Data 3, but for TmY target types. Shown are the top four monosynaptic pathways, the strongest pathway passing through each of the top ten intermediary types (ranking from Extended Data Fig. 7), and the trisynaptic pathway Tm1–TmY–Dm3–TmY (see the section entitled Prediction of spatial normalization). [file 41586_2024_7953_MOESM6_ESM.zip › DataS4/TmY9q/720575940624709373.pdf]

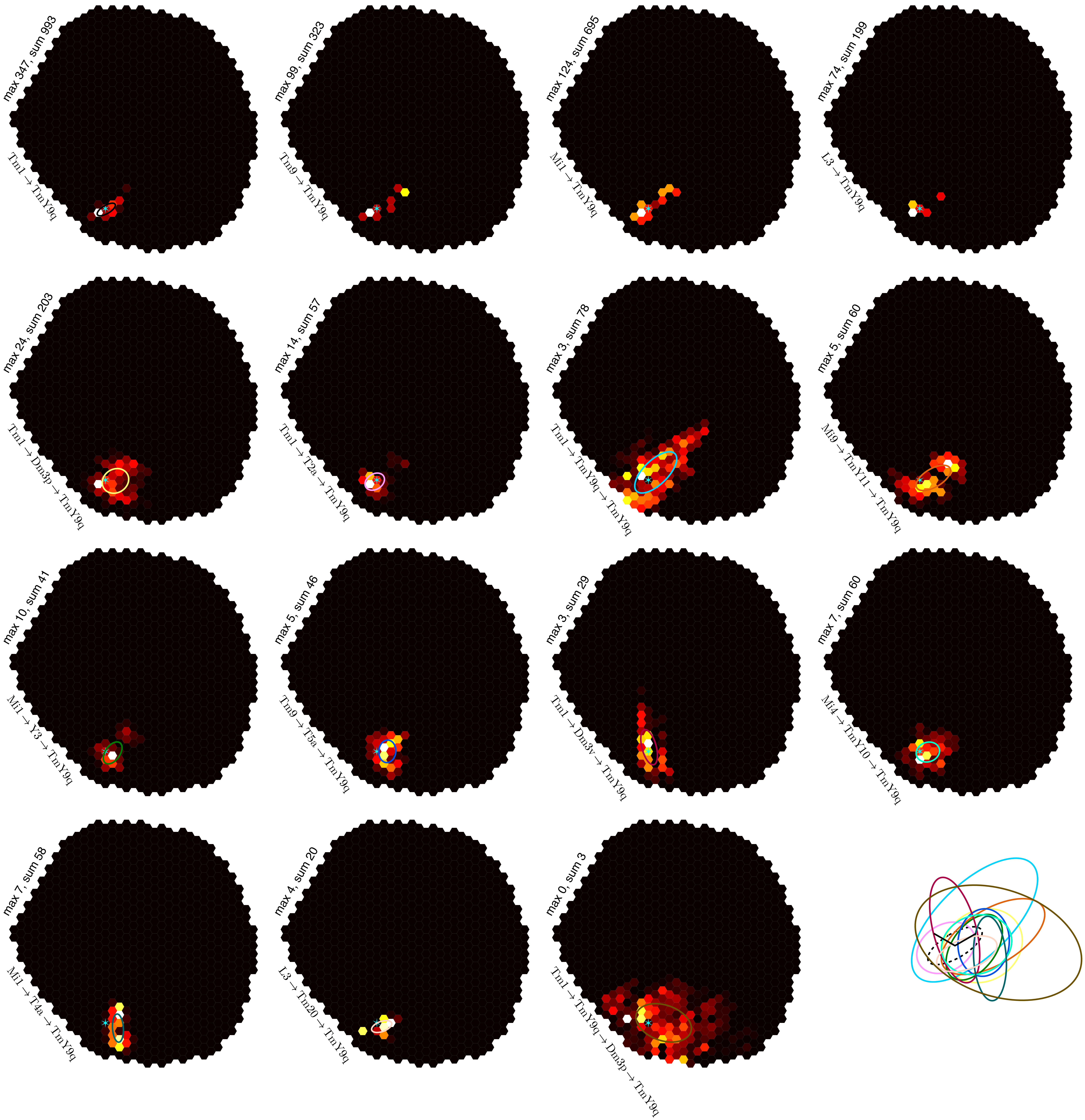
max 347, sum 993
Tm1 → TmY9q
max 99, sum 323
Tm9 → TmY9q
max 124, sum 695
Mi1 → TmY9q
max 74, sum 199
L3 → TmY9q
max 24, sum 203
Tm1 → Dm3p → TmY9q
max 14, sum 57
Tm1 → T2a → TmY9q
max 3, sum 78
Tm1 → TmY9q → TmY9q
max 5, sum 60
Mi9 → TmY11 → TmY9q
max 10, sum 41
Mi1 → Y3 → TmY9q
max 5, sum 46
Tm9 → T5a → TmY9q
max 3, sum 29
Tm1 → Dm3v → TmY9q
max 7, sum 60
Mi4 → TmY10 → TmY9q
max 7, sum 58
Mi1 → T4a → TmY9q
max 4, sum 20
L3 → Tm20 → TmY9q
max 0, sum 3
Tm1 → TmY9q → Dm3p → TmY9q
